# CircPrime: a web-based platform for design of specific circular RNA primers

**DOI:** 10.1186/s12859-023-05331-y

**Published:** 2023-05-19

**Authors:** Fedor Sharko, Golam Rbbani, Prabhugouda Siriyappagouder, Joost A. M. Raeymaekers, Jorge Galindo-Villegas, Artem Nedoluzhko, Jorge M. O. Fernandes

**Affiliations:** 1 Research Center of Biotechnology of the Russian Academy of Sciences, Leninsky Prospect 33/2, 119071 Moscow, Russia; 2 Limited Liability Company ELGENE, Malaya Kalitnikovskaya 16, 109029 Moscow, Russia; 3 National Research Center “Kurchatov Institute”, 1st Akademika Kurchatova Square, 123182 Moscow, Russia; 4 Faculty of Biosciences and Aquaculture, Nord University, Universitetsalléen 11, PB 1490, 8049 Bodø, Norway; 5 Paleogenomics Laboratory, European University at Saint Petersburg, 6/1A Gagarinskaya st., 191187 Saint Petersburg, Russia

**Keywords:** Circular RNAs, RNA-sequencing, circRNAs, Primer design, RT-PCR, qPCR, Validation, PCR, Web platform, Prediction

## Abstract

**Background:**

Circular RNAs (circRNAs) are covalently closed-loop RNAs with critical regulatory roles in cells. Tens of thousands of circRNAs have been unveiled due to the recent advances in high throughput RNA sequencing technologies and bioinformatic tools development. At the same time, polymerase chain reaction (PCR) cross-validation for circRNAs predicted by bioinformatic tools remains an essential part of any circRNA study before publication.

**Results:**

Here, we present the CircPrime web-based platform, providing a user-friendly solution for DNA primer design and thermocycling conditions for circRNA identification with routine PCR methods.

**Conclusions:**

User-friendly CircPrime web platform (http://circprime.elgene.net/) works with outputs of the most popular bioinformatic predictors of circRNAs to design specific circular RNA primers. CircPrime works with circRNA coordinates and any reference genome from the National Center for Biotechnology Information database).

**Supplementary Information:**

The online version contains supplementary material available at 10.1186/s12859-023-05331-y.

## Background

**Fig. 1:**
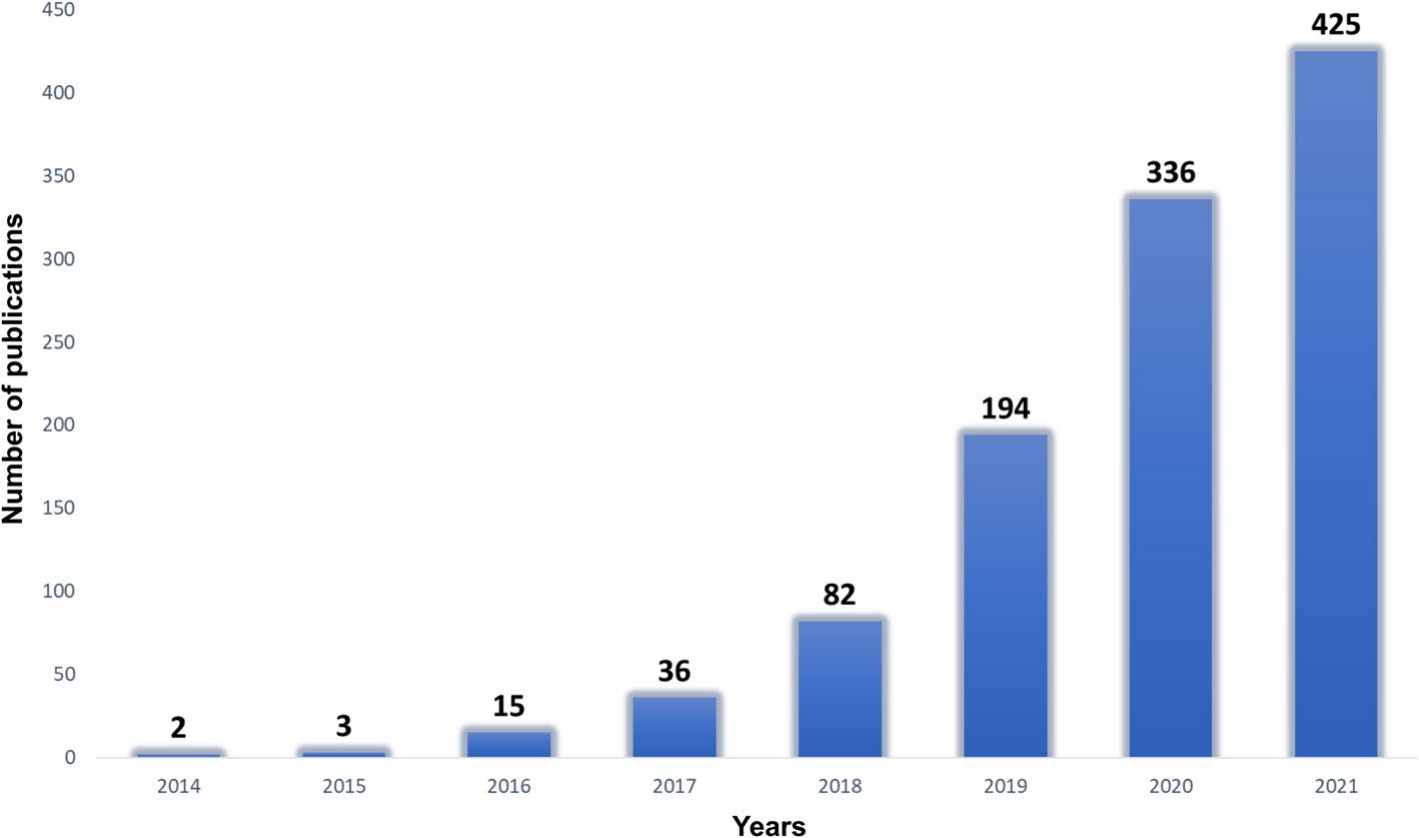
Exponential increase in the number of publications mentioning “circRNA” in their title. X-axis shows years from 2014 until 2021; Y-axis represents the number of publications. *Source*: Web of Science, accessed 6 October 2022

In recent years, there is a marked increase in the number of circular RNA (circRNA)-related studies (Fig. 1). CircRNAs have become a main focus of non-coding RNA biology research because they affect many genetic regulatory networks [15]. These covalently closed-loop RNA molecules are an integral part of the cell regulome and interact with RNA-binding proteins [13, 24]. They can modulate microRNA expression [10, 32] and indirectly affect gene expression [1, 23]. In addition, some of them contain exon parts and can thus be translated into proteins [17, 19]. Recent efforts show that artificial circRNA molecules can be used to enhance peptide production [4].

Modern sequencing technologies make it now possible to identify hundreds of circRNAs that may be used as growth markers for aquaculture [22], as biomarkers and therapeutic targets of human diseases like cancer [16], cardiovascular diseases [2] or brain disorders [7, 20]. However, in silico prediction of circRNAs leads to numerous false-positives [11], as well as inconsistencies among different bioinformatic pipelines [21]. As a result, cross-checking and validation of circRNAs is an essential component of any circRNA study [6].

Reverse transcription PCR (RT-PCR) and quantitative PCR (qPCR) are considered the gold standards for identification of circRNA expression in cells [6]. At the same time, primer design for the circRNAs validation differs from the design for the their linear host genes [28]. To date, only a few tools have been published that allow the development of primers for validation of circRNAs. At the same time, they require additional software to be installed in different operating systems—CircPrimer [35], CircPrimer2.0 [34] and circtools [12], or work as a web tool with already known circRNAs of model organisms, namely human [8] or novel circRNAs for limited number of animal species [28]. Here, to overcome these previous constraints and facilitate the circRNA studies, we present the user-friendly CircPrime web platform (http://circprime.elgene.net/). This tool works with outputs of the most popular bioinformatic predictors of circRNAs, such as CIRI2 [9], KNIFE [26], CIRCexplorer2 [33], find_circ [18], circRNA_finder [30], DCC [5], mapsplice [29] and common BED files. Importantly, CircPrime is suitable for all organisms that have reference genome assemblies in the National Center for Biotechnology Information database (NCBI).

### Implementation

**Fig. 2:**
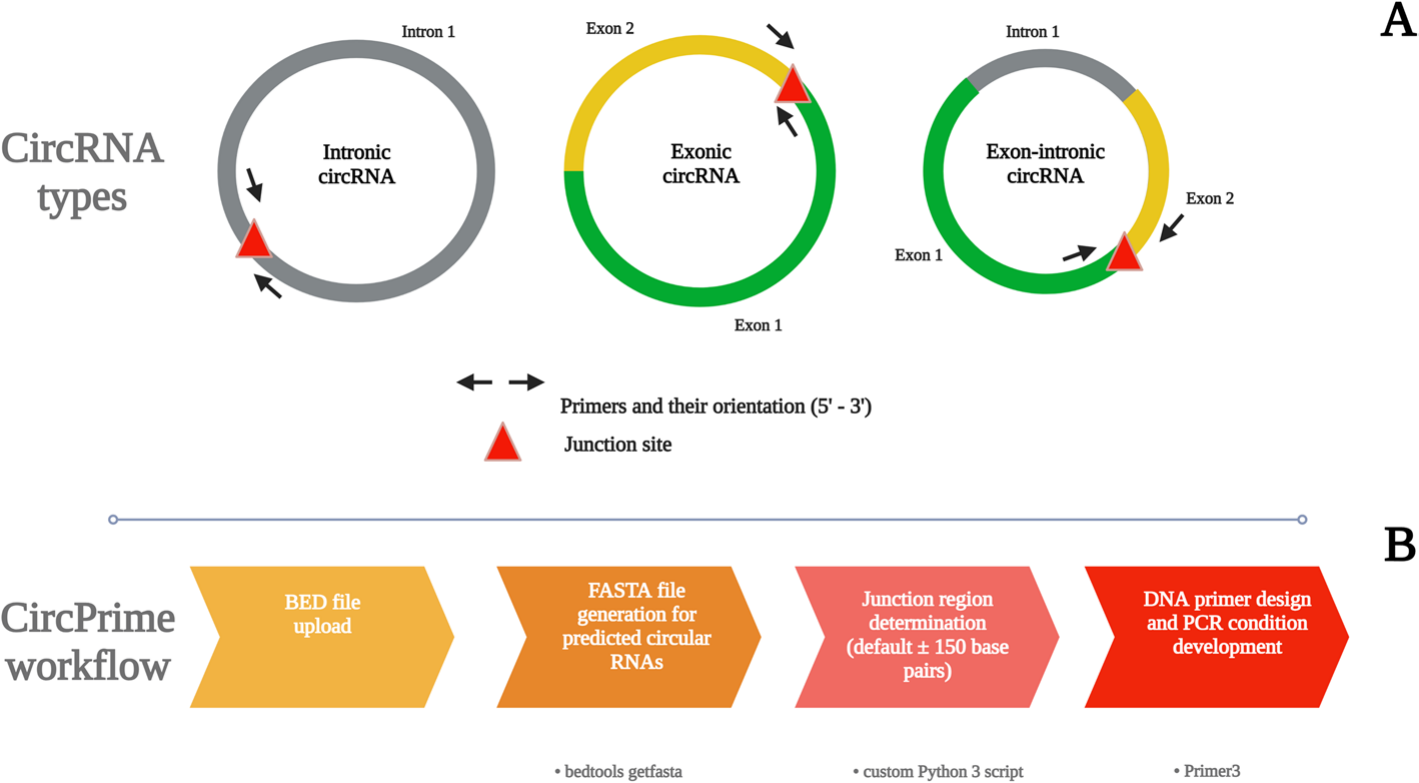
Circular RNA types and overview of the CircPrime pipeline. **A** Possible structural variants of circular RNA for primer design for validation. **B** The main steps of CircPrime pipeline and tools combined in it

To date, there are several methods for PCR-based identification of different circRNAs types (Fig. 2A). One of them is based on rolling circle amplification (RCA). This method avoids deep RNA sequencing and bioinformatic analysis, but is only capable of identifying a limited number of circRNA types [3]. The other most commonly used method assumes a longer workflow, which comprises circRNA enrichment, circRNA-library construction, deep sequencing, circRNA prediction, and finally RT-PCR/qPCR validation of bioinformatically predicted circRNAs [25]. PCR primers for this validation are designed to target the circRNA fragment overlapping a junction (back-splice) site of a specific circRNA (Fig. 2A).

We developed CircPrime, as a streamlined pipeline in Python 3 and web platform, which makes use of output files from the most popular circRNAs in silico predictors. The web application uses the flask framework (https://github.com/pallets/flask). The representative state transfer (REST) was chosen as the architecture for the design of the web platform [31], which fully complies with the HTTP protocol. The CircPrime script implemented into the web platform currently contains the four main modules shown in Fig. 2B and works under the parameters presented in Table 1.

**Table 1:** Required and default parameters for CircPrime web platform usage

CircPrime parameter	Parameter description
CircRNA BED file	Required
Shift range left	Default 150 nucleotides
Shift range right	Default 150 nucleotides
Optimum length of a primer	Default 19 nucleotides
Minimum acceptable length of a primer	Default 15 nucleotides
Maximum acceptable length of a primer	Default 20 nucleotides
PCR product size	Default 150–200 nucleotides
Optimum melting temperature for a primer	In Celsius
Minimum acceptable melting temperature for a primer	In Celsius
Maximum acceptable melting temperature for a primer	In Celsius
Number of primer sets	Default 4 sets

After the first step, which includes BED file uploading, CircPrime generates FASTA files using circRNA coordinates and reference genome from the NCBI. Then CircPrime extracts junction regions from the uploaded BED file and develops primer sets with the recommended melting temperature (Tm) for each circRNA in the list (up to 100) using Primer3 (Fig. 2B) [14, 27]. An example of the CircPrime output is presented as Supplementary Dataset 2.

## Results

The novel CircPrime web-based platform was evaluated to design primer sets for RT-PCR validation of circRNA expression in the muscle transcriptome of a teleost, the Nile tilapia (*Oreochromis niloticus*). Successfully, we showed that CircPrime significantly simplifies the primer design process for bioinformatically predicted circRNAs without the need to upload a reference genome of the organism studied. In this study, we applied circRNA transcriptome sequencing and circRNA prediction by CIRI2 [9] in four Nile tilapia skeletal muscle tissue specimens for successful validation of the novel CircPrime web platform. This tool designed circRNA primer pairs, which were used to validate their RT-PCR efficiency. The genome coordinates for 10 circular RNAs expressed in all muscle samples were used for primer design. CircPrime was able to design primer sets for 9 of the 10 circRNAs (Supplementary Dataset 2). Subsequently, four of them (Table S1) were validated using PCR (see details in Supplementary Material).

At present, circPrimer [35], circPrimer2.0 [34], and circtools [12] are mainly used for primer design, and all of them cope well with their main task. At the same time, these tools have a number of significant differences in the interface and functionality, e.g., circtools [12] is a modular platform based on the Python3, which combines several functions in a single script managed from the command line. Circtools has numerous parameters and allows to choose various options for primer design. CircPrimer [35] and its updated version, CircPrimer2 [34], are convenient tools for circRNA research that are implemented as a graphical interface and a command-line interface. CircPrimer2 allows users to search, annotate, and visualize circRNAs and also helps users develop primers for circRNAs and verify the specificity of circRNA primers. Unlike circPrimer [35], circPrimer 2.0 [34], and circtools [12], which are pre-installed to work only with the mouse and human genomes, CircPrime itself determines the genome by ID using the NCBI database, and its web interface does not need to be installed and is immediately ready for convenient and fast use.

We expect that this bioinformatic tool will play a relevant role on varied studies describing circRNAs expression and their possible functionality. CircPrime is applicable for any organism, including even those with a relatively poorly annotated genome assembly, such as Nile tilapia (NCBI accession: GCF_001858045.2_O_niloticus_UMD_NMBU).

## Conclusions

Herein, we present a Circprime web platform (http://circprime.elgene.net/) for PCR primer design and PCR conditions development for validation of circRNAs predicted based on RNA-sequencing data using different types of bioinformatics tools. We expect that this web tool will be convenient for users who intend to analyze the expression of circRNAs in animal and plant transcriptomes.

### Availability and requirements

Project name: CircPrime. Project home page: http://circprime.elgene.net/. CircPrime documentation: https://circprime.readthedocs.io/. Operating system(s): Platform independent. Programming language: Python 3.10. Other requirements: None. License: GNU GPL Version 3.

## Supplementary Information

**Additional file 1.** **Supplementary Dataset 1:** CIRI2 output files for four Nile tilapia circRNA-Seq datasets. Overlapped circRNAs. Overlapped and overrepresented circRNAs of Nile tilapia muscle, which were used for CircPrime web-based platform validation.

**Additional file 2.** **Supplementary Dataset 2:** An example of CircPrime output: primer pairs and PCR conditions for them, which were developed for bioinformatically predicted circRNAs.

**Additional file 3.** **Supplementary File:** Additional material that supports the main manuscript. Extended Methods and Results sections.

## Data Availability

The user-friendly CircPrime tool for circular RNA primer development is written in Python 3 and implemented on a web-based platform. It is freely available online at http://circprime.elgene.net/. The RNA-seq dataset generated and analysed during the current study is available in the GEO (NCBI) repository, under the accession number PRJNA826285: https://www.ncbi.nlm.nih.gov/bioproject/PRJNA826285 (accessed on 05 May 2023). CircPrime documentation is available at https://circprime.readthedocs.io/.

## Abbreviations

PCR: Polymerase chain reaction
CircRNA: Circular RNA
RT-PCR: Reverse transcription PCR
qPCR: Quantitative PCR
NCBI: National Center for Biotechnology Information database
RCA: Rolling circle amplification
